# The relationships between fat-soluble vitamin deficiency and childhood obesity risk

**DOI:** 10.3389/fped.2026.1782635

**Published:** 2026-05-20

**Authors:** Fang Zhou, Xiaofang Li, Li Zhu, Zhenzhen Jin, Hengdong Fu, Zhouying Guo, Yu Fang

**Affiliations:** 1 Department of Paediatrics, Traditional Chinese Medical Hospital of Zhuji, Zhejiang, China; 2 Dispensary of Traditional Chinese Medicine, Traditional Chinese Medical Hospital of Zhuji, Zhejiang, China; 3 Wenzhou Hospital of Integrated Traditional Chinese and Western Medicine Affiliated to Zhejiang Chinese Medical University, Zhejiang, China

**Keywords:** childhood obesity, fat-soluble vitamins, lifestyle, vitamin A, vitamin E, vitamin K

## Abstract

**Objective:**

This study aimed to examine the associations between serum levels of fat-soluble vitamins (A, D, E, and K) and obesity-related indicators in children.

**Methods:**

A cross-sectional study was conducted involving 208 children stratified into an obese cohort (*n* = 104) and a control group (*n* = 104). Serum concentrations of vitamins A, D, E, and K were measured via liquid chromatography-tandem mass spectrometry (LC-MS), a highly sensitive and specific method for vitamin quantification. Season of blood collection was recorded and categorized as summer/autumn or winter/spring. Age-stratified analyses were conducted to explore associations between vitamin levels and obesity indices, including BMI, body fat percentage, and waist-to-height ratio.

**Results:**

Vitamin D deficiency was significantly more prevalent in the obese group (62.50% vs. 28.85%, *P* < 0.001). Age-stratified analysis showed that vitamin D deficiency was associated with obesity in children aged 6–11 years (OR = 3.329, 95% CI: 1.618–6.854) and adolescents aged 11–17 years (OR = 4.890, 95% CI: 2.505–9.542).

**Conclusion:**

Findings highlight a substantial association between fat-soluble vitamin deficiency and pediatric obesity.

## 1 Introduction

Childhood obesity has escalated into a critical global public health emergency, with current estimates indicating that approximately 38.2 million children aged below five years are affected by excess weight or obesity globally. Additionally, the prevalence of excess weight extends to older age groups, with approximately 340 million children and adolescents aged between five and eighteen years also classified as overweight or obese globally (1). A recent global meta-analysis by Zhang et al. (2), covering 154 countries and over 45.8 million children, reported a global obesity prevalence of 8.5% (95% CI: 8.2%–8.8%), with substantial regional variation (0.4%–28.4%). Notably, the prevalence of obesity increased 1.5-fold from 2000–2011 to 2012–2023, highlighting the escalating nature of this epidemic (2). With the shift in dietary patterns and reduced physical activity, the prevalence of overweight problems in minors continues to climb. The ramifications of obesity extend far beyond mere weight concerns, exerting a profound impact on both the physiological growth trajectory and psychological well-being of children. Notably, this condition markedly elevates the susceptibility to chronic ailments in later life, such as metabolic disorders, cardiovascular pathologies, and type 2 diabetes mellitus.

The circulating concentrations of fat-soluble vitamins—encompassing vitamins A, E, D, and K—are intricately linked to an array of factors, including feeding modalities, dietary compositions, and an individual's inherent metabolic efficiency, and deficiencies or excesses of any one of these elements can lead to illness and even death in children. Research endeavors have illuminated the pivotal role of adipocytes in orchestrating inflammatory responses within the body. These specialized immune cells release a spectrum of pro-inflammatory cytokines via both receptor-mediated and non-specific secretory pathways (3, 4), which puts obese children in a state of chronic inflammation (5). Therefore, revealing the underlying pathogenic mechanisms and associated risk factors is crucial for developing effective prevention and control strategies.

Most existing studies have focused on vitamin D alone, while comprehensive assessments of all four fat-soluble vitamins (A, D, E, K) using serum biomarkers—particularly in relation to childhood obesity—remain limited. However, dietary patterns observed among obese children often deviate from this balanced intake. These children tend to consume an excess of high-calorie foods while exhibiting a relative aversion to fruits and vegetables, thereby disrupting the nutritional equilibrium crucial for their growth and development, and thus may have an insufficient vitamin A intake (6). Due to the above reasons, fat-soluble vitamin levels in children in China are currently generally deficient. In 2021, a research investigation conducted by Shandong Hospital delved into the vitamin D levels in children under six living in Jinan. This epidemiological study found that serum vitamin D concentrations among preschoolers in Jinan closely paralleled those observed in their peers in Guangzhou and even marginally surpassed the levels reported among Turkish children of a similar age group and that the average vitamin D level of preschoolers had not yet reached an adequate level, with the deficiency and insufficiency rates as high as 61.79% (7). In addition, there is a similar research study in Hangzhou. Hangzhou Obstetrics and Gynecology Hospital investigated the fat-soluble vitamin levels in 3,613 cases of children aged 2–6 years old, and further analysis of the data yielded gender-specific disparities in vitamin A and D deficiencies. Notably, male children exhibited a higher prevalence of vitamin A deficiency than females. In Hangzhou, a distinct locality, the rate of vitamin A deficiency was documented at 0.25%, whereas the proportion of children with suboptimal vitamin A levels (insufficiency) reached 10.02%. In contrast, girls had a higher incidence of vitamin D insufficiency than boys. The combined prevalence of vitamin D deficiency and insufficiency in the study population was 0.42% and 2.66%, respectively. The deficiency in spring and winter is higher than that in summer and fall, and vitamin D deficiency is also serious with age (8). And there is no similar survey of all-age fat-soluble vitamin levels for the Zhuji area.

This study aimed to examine the associations between serum levels of fat-soluble vitamins (A, D, E, K) and obesity-related indicators in children. To achieve this, the study will undertake a comprehensive assessment of vitamin status by juxtaposing the serum/plasma vitamin concentrations of children categorized within an obese cohort against those of a control group (herein referred to as the “reference group” to avoid ambiguity with the term “control group”, which lacks specificity in scientific contexts), and the correlation between vitamin levels and obesity indicators will be further explored. In addition, this investigation will scrutinize the correlation between vitamin deficiencies and obesity across various pediatric age brackets and the independent effect of vitamin deficiency on the risk of obesity in children. In addition, the research endeavors to catalyze the dissemination and adoption of health-promoting lifestyles among children, thereby fostering long-term improvements in nutritional status and obesity prevention.

## 2 Data and methods

### 2.1 Study subjects

Children were recruited from Zhuji City, Zhejiang Province, China. In alignment with the diagnostic guidelines for pediatric obesity established by the World Health Organization (WHO), the study cohort was partitioned into an obese group and a control group (normal weight), each comprising 104 participants. Children categorized within the obese group exhibited body mass index (BMI) values exceeding the 95th percentile threshold for their respective age groups, while the BMI of children in the control group was between the 5th and 85th percentiles of BMI of children of the same age. This study was approved by the Ethics Committee of Traditional Chinese Medical Hospital of Zhuji (Approval No. 2023019). Written informed consent was obtained from the parents or legal guardians of all participating children.

### 2.2 Research methodology

#### 2.2.1 General information survey

The research team designed a detailed questionnaire for collecting general information about the children and their families. The questionnaire included sociodemographic information such as children's age, gender, date of birth, home address, parents' education level, and family income. In addition, children were asked about their lifestyles, such as cumulative daily exposure to screen-based media (encompassing television viewing and engagement with digital devices like computers, smartphones, and tablets), weekly exercise time (including school physical education classes, extracurricular physical activities, etc.), and dietary habits (e.g., whether or not they ate meals regularly, whether or not they had a preference for high-calorie foods, etc.). The questionnaires were completed by trained enumerators who interviewed the children's parents face-to-face to ensure the accuracy and completeness of the information. During the survey, the enumerators explained each item in the questionnaire in detail to ensure that parents understood and answered accurately. For some vague or inconsistent answers, the investigator will further pursue and verify them to ensure data quality.

#### 2.2.2 Physical examination

All children undergo a standardized physical examination, completed by a team of experienced pediatricians and nurses. The physical examination includes measurement of height, weight, and waist and hip circumference. Anthropometric assessments were conducted with precision: vertical stadiometers calibrated to a resolution of 0.1 cm (Seca 213, Seca, Hamburg, Germany) were employed for height measurement, while electronic weighing scales with a sensitivity of 0.1 kg (Tanita BC-418, Tanita, Tokyo, Japan) recorded body mass. During anthropometric assessment, participants were instructed to remove footwear, socks, and bulky outerwear, adopting a standardized upright posture. Waist circumference was measured horizontally at a site 1 cm above the umbilicus, while hip circumference was recorded at the maximal protrusion of the gluteal region using a non-elastic, flexible tape calibrated to 0.1 cm precision. Height and weight were used to determine BMI, while body fat percentage was estimated via bioelectrical impedance analysis (BIA), a validated, non-invasive, and rapid method for pediatric adiposity quantification. Prior to deployment, all measurement instruments underwent mandatory calibration protocols to guarantee the fidelity of quantitative outcomes. During the physical examination, the medical staff strictly followed the operational protocols to ensure the reliability and reproducibility of the measurement data.

#### 2.2.3 Vitamin testing

Vitamin assay is one of the key aspects of this study. Fasting venous blood specimens were procured from pediatric participants, and concentrations of fat-soluble vitamins (A, D, E, and K) were measured using ultra-high-performance liquid chromatography coupled with tandem mass spectrometry (LC-MS) using an AB Sciex QTRAP 6500+ LC-MS/MS system (AB Sciex, Framingham, MA, USA), leveraging its superior selectivity and sensitivity for complex metabolite profiling, a technique renowned for its sensitivity, specificity, and analytical precision in clinical biochemical profiling. LCMS offers exceptional selectivity and sensitivity in the analysis of complex metabolite profiles; this technology is renowned for its high sensitivity, high specificity, and high analytical accuracy in clinical biochemistry. The date of blood collection was recorded for each participant, and season was categorized as summer/autumn (June–November) or winter/spring (December–May), based on the seasonal variation in sunlight exposure known to influence vitamin D levels. Venous blood sampling was conducted exclusively by certified nursing personnel, with specimens promptly transferred into anticoagulant-treated tubes post-collection and expedited to the laboratory for analysis. Each analytical method employed underwent stringent validation to uphold the analytical rigor and reproducibility of results, ensuring alignment with accredited laboratory standards for diagnostic accuracy. Laboratory staff are professionally trained to perform the tests in strict compliance with the operating procedures, while regular indoor and inter-room quality control is performed to guarantee the precision and consistency of the test results.

### 2.3 Statistical analysis

Data were analyzed using SPSS 26.0 statistical software. The data were first tested for normality; continuous variables adhering to a normal distribution were summarized ($\bar{x}\pm s$), and intergroup discrepancies were statistically assessed with an independent samples *t*-test. For continuous data exhibiting non-normal distribution, the median (interquartile range, IQR) [M(IQR)] was employed as the descriptive statistic, and pairwise comparisons were performed using the Mann–Whitney *U*-test. Categorical data were presented as counts (along with their proportional percentages), and intergroup disparities were examined via the chi-square test, based on anticipated cell frequencies and adherence to sample size criteria.

For correlation assessments, the Pearson or Spearman correlation coefficient was employed to quantify the magnitude and direction of relationships between vitamin biomarker levels and obesity parameters. In age-stratified subgroup analyses, logistic regression was utilized to model the odds of obesity associated with vitamin deficiency, with regression coefficients (β), standard errors (SE), Wald chi-square statistics (Wald), odds ratios (OR), and their corresponding 95% confidence intervals (95% CIs) derived. Statistical significance was evaluated using two-tailed hypothesis testing, with a *P*-value threshold of <0.05 deemed indicative of meaningful associations.

A multivariate logistic regression model was used to evaluate the independent effects of vitamin deficiency, parental obesity, and lifestyle factors to pediatric obesity risk, with obesity status designated as the outcome variable. Covariate adjustment for potential confounders—including age, gender, and household socioeconomic status (SES) and season of blood collection (summer/autumn vs. winter/spring) —was implemented to mitigate residual confounding. Adjusted ORs and their 95% CIs were calculated to quantify the isolated effects of each predictor on obesity likelihood, adhering to rigorous statistical protocols to safeguard the validity and interpretability of findings. All inferential procedures adhered to established methodological standards, ensuring robustness against Type I/II errors and promoting replicability of results.

## 3 Results

### 3.1 Comparison of baseline characteristics

No significant differences were observed in age or sex distribution between groups. However, the obese group had significantly lower household income, higher parental BMI, greater daily screen time, and less weekly exercise (all *P* < 0.001) (Table 1).

**Table 1:** Comparison of baseline characteristics.

Indicators	Obese group (*n* = 104)	control group (*n* = 104)	Test value (*t*/χ²/*Z*)	*P*-value
Age (years, $\bar{x}\pm s$)	8.36 ± 1.24	8.41 ± 1.17	*t* = 0.327	0.744
Male [cases, *n* (%)]	53 (50.96%)	52 (50.00%)	χ² = 0.019	0.891
Family income [$10,000/year, median (IQR)]	5.2 [3.8–6.7]	6.8 [5.1–8.3]	*Z* = 4.127	<0.001
Father BMI (kg/m², $\bar{x}\pm s$)	26.73 ± 3.12	23.84 ± 2.67	*t* = 6.892	<0.001
Mother BMI (kg/m², $\bar{x}\pm s$)	25.68 ± 2.94	22.97 ± 2.51	*t* = 6.245	<0.001
Daily screen time (hours, $\bar{x}\pm s$)	3.72 ± 1.28	2.13 ± 0.97	*t* = 9.634	<0.001
Weekly exercise time (hours, $\bar{x}\pm s$)	2.31 ± 1.12	3.82 ± 1.49	*t* = 8.127	<0.001

### 3.2 Comparison of vitamin deficiency prevalence

The prevalence of deficiencies in fat-soluble vitamins (A, D, E, and K) was significantly higher in the obese group compared to the control group. Vitamin D deficiency reached 62.50% in the obese group (compared to 28.85% in the control group, *P* < 0.001), while vitamin A deficiency was 42.31% (18.27% in the control group, *P* < 0.001). The incidence of combined deficiencies (≥3 types) was 32.69% in the obese group (8.65% in the control group, *P* < 0.001) (Table 2).

**Table 2:** Comparison of prevalence of vitamin deficiency.

Type of vitamin deficiency	Obese group (*n* = 104)	control group (*n* = 104)	*χ*² value	*P*-value
Vitamin A deficiency (<0.3 μg/ml)	44 (42.31%)	19 (18.27%)	18.215	<0.001
Vitamin D deficiency (<20 ng/ml)	65 (62.50%)	30 (28.85%)	25.738	<0.001
Vitamin E deficiency (<5 μg/ml)	37 (35.58%)	15 (14.42%)	12.647	<0.001
Vitamin K deficiency (<0.5 ng/ml)	29 (27.88%)	11 (10.58%)	9.843	0.002
Combined deficiency* (≥3)	34 (32.69%)	9 (8.65%)	18.932	<0.001

Combined deficiency*: defined as simultaneous deficiency of 3 or more fat-soluble vitamins. Deficiency cutoffs were defined according to established clinical guidelines: vitamin A < 0.3 μg/ml, vitamin D < 20 ng/ml, vitamin E < 5 μg/ml, vitamin K < 0.5 ng/ml.

### 3.3 Associations between vitamin concentrations and obesity-related metrics

Table 3 showed that, vitamin D demonstrated a robust negative correlation with both BMI (*r* = −0.42, *P* = 0.003) and body fat percentage (*r* = −0.37, *P* = 0.008). Similarly, vitamin A exhibited a statistically significant inverse association with BMI (*r* = −0.38, *P* = 0.005) and body fat percentage (*r* = −0.32, *P* = 0.015), but the association of vitamins E and K with obesity indicators was weak, and some of them did not reach statistical significance (*P* > 0.05).

**Table 3:** Correlation between vitamin levels and obesity indicators.

Vitamin type	BMI (kg/m²)	Body fat percentage (%)	Waist-to-height ratio
Vitamin A (μg/ml)	*r* = −0.38 (*P* = 0.005)	*r* = −0.32 (*P* = 0.015)	*r* = −0.25 (*P* = 0.048)
Vitamin D (ng/ml)	*r* = −0.42 (*P* = 0.003)	*r* = −0.37 (*P* = 0.008)	*r* = −0.29 (*P* = 0.032)
Vitamin E (μg/ml)	*r* = −0.27 (*P* = 0.041)	*r* = −0.21 (*P* = 0.093)	*r* = −0.18 (*P* = 0.164)
Vitamin K (ng/ml)	*r* = −0.19 (*P* = 0.141)	*r* = −0.22 (*P* = 0.058)	*r* = −0.16 (*P* = 0.203)

### 3.4 Age-stratified analysis

Age-stratified logistic regression modeling was employed to evaluate the link between low vitamin D levels and the risk of obesity in children (see Tables 4 and 5 for details). Results demonstrated an age-dependent escalation in the magnitude of association, with the earliest detectable effect observed in lower elementary school children (3–6 years old), resulting in an adjusted OR of 2.210 (95% CI: 1.016–4.807, *P* = 0.046); in children in the upper elementary school grades (6–11 years old), this association was further enhanced, with an OR of 3.329 (95% CI: 1.618–6.854, *P* = 0.001); by adolescence (11–17 years), the strongest association emerged among children aged 7–12 years, characterized by an aOR of 4.890 (95% CI: 2.505–9.542, *P* < 0.001), reflecting a 389% elevated obesity risk in vitamin D-deficient participants relative to those with adequate levels. In contrast, no statistically significant association was detected among preschool-aged children (0–3 years; *P* = 0.285).

**Table 4:** Variable types and assignment rules.

Variable type	Assignment
Dichotomous variables	(1) Vitamin D deficiency: 0 = normal (≥20 ng/ml), 1 = deficient (<20 ng/ml)
	(2) Obesity in both parents: 0 = none, 1 = both parents BMI ≥25 kg/m²
Continuous variables	(3) Age (years): raw values, ungrouped
	(4) Household income ($10,000/year): entered as actual values, median and quartiles described
Ordered/unordered categorical variables	(5) Age group: preschool (0–3 years) = 1, lower primary (3–6 years) = 2, upper primary (6–11 years) = 3, adolescence (11–17 years) = 4

**Table 5:** Age-stratified analysis: vitamin D deficiency and obesity association (logistic regression).

Age group	Regression coefficient (β)	Standard error (SE)	Wald chi-square value (Wald)	OR (95% CI)	*P*-value
Preschool (0–3 years)	0.482	0.451	1.142	1.621 (0.670–3.915)	0.285
Lower primary (3–6 years)	0.794	0.396	4.013	2.210 (1.016–4.807)	0.046
Upper primary (6–11 years old)	1.203	0.369	10.615	3.329 (1.618–6.854)	0.001
Adolescence (11–17 years old)	1.588	0.341	21.732	4.890 (2.505–9.542)	<0.001

### 3.5 Multifactorial regression model

In the multifactorial regression model, after adjusting for confounders such as age, gender, and household income, vitamin D deficiency (OR = 3.211, 95% CI: 1.819–5.670, *P* = 0.002), and season of blood collection (summer/autumn vs. winter/spring), vitamin D deficiency (OR = 2.713, 95% CI: 1.336–5.509, *P* = 0.006), multivitamin deficiency (OR = 3.889, 95% CI: 2.138–7.051, *P* < 0.001), obesity in both parents (OR = 2.780, 95% CI: 1.420–5.431, *P* = 0.008), >2 h of daily screen time (OR = 1.941, 95% CI: 1.117–3.358, *P* = 0.026), and <3 h of weekly exercise (OR = 2.152, 95% CI: 1.240–3.729, *P* = 0.011) were identified as independent risk factors for pediatric obesity. Additionally, season of blood collection (winter/spring vs. summer/autumn) was significantly associated with obesity risk (OR = 1.844, 95% CI: 1.028–3.307, *P* = 0.040) (Table 6).

**Table 6:** Multifactorial regression model: predictors of obesity risk (adjusted for age, sex, household income).

Variable	Regression coefficient (β)	Standard error (SE)	Wald chi-square value (Wald)	Adjusted OR (95% CI)	*P*-value
Vitamin A deficiency	1.218	0.372	10.745	3.380 (1.635–6.985)	0.001
Vitamin D deficiency	1.166	0.358	10.607	3.211 (1.819–5.670)	0.002
Multivitamin deficiency	1.357	0.327	17.283	3.889 (2.138–7.051)	<0.001
Obesity in both parents	1.023	0.374	7.472	2.780 (1.420–5.431)	0.008
Daily screen time >2 h	0.663	0.291	5.189	1.941 (1.117–3.358)	0.026
Exercise time <3 h per week	0.765	0.297	6.643	2.152 (1.240–3.729)	0.011
Season of blood collection (winter/spring vs. summer/autumn)	0.612	0.298	4.218	1.844 (1.028–3.307)	0.040

## 4 Discussion and analysis

### 4.1 Mechanism discussion

In this study, the prevalence of vitamin A deficiency was substantially elevated among children in the obese cohort, and serum vitamin A levels were negatively correlated with obesity indicators. Mechanistically, vitamin A regulates retinol-binding protein synthesis, which plays a key role in lipid metabolism in adipocytes, and its deficiency may be associated with chronic low-grade inflammation that affects adipose tissue metabolism (9).

A significantly elevated prevalence of vitamin D deficiency was observed in the obese cohort compared to the control group. Mechanistically, vitamin D insufficiency impairs intestinal calcium absorption, which activates parathyroid hormone (PTH) and promotes fat synthesis in adipocytes. Additionally, vitamin D deficiency is linked to sustained low-grade inflammation, a key driver of obesity pathogenesis (10).

The prevalence of vitamin E deficiency was significantly higher in the obese group compared to the control group. Mechanistically, vitamin E depletion may induce redox imbalance by promoting reactive oxygen species (ROS) accumulation, triggering oxidative stress that exacerbates adipocyte hypertrophy, inflammation, and insulin resistance (11).

A markedly elevated prevalence of vitamin K deficiency was found among obese children compared to controls, with serum vitamin K levels inversely correlated with adiposity parameters. Mechanistically, vitamin K deficiency may compromise adipocyte perfusion via dysregulated matrix Gla protein (MGP) carboxylation and vascular calcification, and may also perturb adipocyte function through impaired osteocalcin-mediated bone-adipose crosstalk (12).

### 4.2 Analysis of results

Regarding the prevalence of vitamin deficiency, this study revealed a substantially elevated prevalence of concurrent deficiencies in vitamins A, D, E, and K among pediatric obesity cases compared to non-obese controls, which suggests that vitamin deficiency may be an important risk factor for childhood obesity. Vitamin deficiency may lead to disorders of fat metabolism, which in turn promotes the development of obesity. However, given that household income was significantly lower in the obese group (*P* < 0.001), it remains plausible that vitamin deficiency may partly reflect broader socioeconomic disparities in dietary quality rather than serving as an independent causal factor. The multifactorial regression model adjusted for income, yet residual confounding by unmeasured aspects of socioeconomic status cannot be entirely excluded. In addition, insufficient vitamin intake could amplify the progression of obesity by provoking chronic inflammatory responses and oxidative stress, thereby impairing adipocyte metabolic functions (13).

Correlation analyses revealed robust inverse associations between vitamin A and vitamin D and indices of adiposity (e.g., BMI *z*-scores, waist-to-height ratio). In contrast, the observed weak correlations between α-tocopherol (vitamin E) and phylloquinone/menaquinone (vitamin K) levels and obesity biomarkers may be attributed to insufficient statistical power secondary to limited cohort size or potential analytical variability introduced by assay sensitivity thresholds and pre-analytical sample handling artifacts.

Age-stratified examinations revealed notable disparities in the linkage between vitamin D insufficiency and pediatric obesity across various age cohorts. Specifically, the correlation was more pronounced among children in the lower and upper primary school grades, as well as adolescents, whereas it appeared weaker in preschool-aged children. This implies that the impact of vitamin D deficiency on obesity could be linked to age. Children's growth and development are faster in the lower primary grades, upper primary grades, and puberty, which increases the need for vitamin D. Vitamin D deficiency may lead to disorders of fat metabolism, which in turn promotes the development of obesity (14).

In a literature review of fat-soluble vitamins, Nemati et al. (15) investigated potential associations between the metabolism of these vitamins and obesity, which is consistent with the results of the functional and metabolic changes of fat-soluble vitamins in obese populations that were explored in this study.

### 4.3 Seasonal variation in vitamin D and obesity risk

In the present study, season of blood collection was significantly associated with obesity risk in the multifactorial regression model, with children sampled during winter/spring showing higher odds of obesity compared to those sampled during summer/autumn (OR = 1.844, 95% CI: 1.028–3.307, *P* = 0.040). This finding is consistent with the well-established seasonal variation in vitamin D status, as sunlight exposure—the primary source of vitamin D synthesis—is substantially lower during winter and spring months. Previous studies have documented that vitamin D levels typically peak in late summer and reach their nadir in late winter/early spring (16). The observed seasonal effect may therefore partly reflect the concurrent decline in vitamin D levels during colder seasons, which itself has been associated with increased adiposity and metabolic dysregulation. However, it is important to note that seasonal factors may also influence other obesity-related behaviors, such as outdoor physical activity and dietary patterns. Children tend to engage in less outdoor exercise during winter months, which may independently contribute to weight gain. Although we adjusted for season of blood collection in the regression model, the interplay between seasonal variations in vitamin D, physical activity, and dietary intake remains complex and warrants further investigation in longitudinal studies.

### 4.4 Public health implications

First, by increasing the level of vitamin intake in children, the occurrence of childhood obesity can be effectively prevented and controlled. Second, improving family economic status and improving children's dietary structure; strengthening parental health education to reduce the incidence of parental obesity; and encouraging children to reduce screen time and increase exercise time. Finally, for children in the lower elementary grades, upper elementary grades, and adolescents, focused monitoring and supplementation of vitamin D are needed to prevent and control the onset of obesity.

### 4.5 Limitations

While the present investigation has advanced understanding of the link between suboptimal fat-soluble vitamin status and pediatric obesity, several methodological constraints warrant attention. Firstly, the cross-sectional design precluded causal inference, necessitating longitudinal cohort studies or randomized controlled trials to clarify causality between vitamin deficiencies and childhood obesity. Secondly, data collection spanned from June to March, covering both summer/winter seasons; seasonal variation is known to significantly influence vitamin D levels due to differences in sunlight exposure. Although we adjusted for season of blood collection in the multivariable regression model, residual confounding from unmeasured seasonal factors (e.g., outdoor activity patterns, dietary changes) cannot be fully excluded. Thirdly, the limited sample size may constrain the generalizability of the findings, requiring replication in larger populations. Finally, this study focused exclusively on quantifying serum/plasma levels of vitamins A, D, E, and K, neglecting assessments of other critical micronutrients (e.g., B-complex vitamins, minerals) that may synergistically influence obesity risk.

## 5 Summary

In summary, this research explored the connection between insufficient multivitamin consumption and pediatric obesity, identifying vitamin deficiency as a significant risk factor. Vitamin deficiency may be associated with obesity by affecting adipocyte metabolic processes, being associated with chronic inflammation and oxidative stress. Therefore, there is a need to strengthen the monitoring and intervention of vitamin intake and improve children's lifestyles in order to prevent and control the occurrence of childhood obesity. Subsequent investigations should augment sample sizes and employ cohort studies or randomized controlled trials to elucidate the causal mechanisms linking vitamin deficiency to childhood obesity.

## Data Availability

The raw data supporting the conclusions of this article will be made available by the authors, without undue reservation.

## References

[1] World Health Organization. Obesity and overweight [EB/OL]. (2021-06-09) [2021-09-06].

[2] Zhang X Liu J Ni Y Yi C Fang Y Ning Q Global prevalence of overweight and obesity in children and adolescents: a systematic review and meta-analysis. JAMA Pediatr. (2024) 178(8):800–13. 10.1001/jamapediatrics.2024.1576 38856986 PMC11165417

[3] Gasmi A Noor S Menzel A Doşa A Pivina L Bjørklund G. Obesity and insulin resistance: associations with chronic inflammation, genetic and epigenetic factors. Curr Med Chem. (2021)) 28(4):800–26. 10.2174/0929867327666200824112056 32838708

[4] Gavaldà-Navarro A Villarroya J Cereijo R Giralt M Villarroya F. The endocrine role of brown adipose tissue: an update on actors and actions. Rev Endocr Metab Disord. (2022) 23(1):31–41. 10.1007/s11154-021-09640-6 33712997

[5] Zou Y Zhang RH Huang LC Zhao D Su DT Meng J Serum levels of vitamin D, retinol, zinc, and CRP in relation to obesity among children and adolescents. Eur J Med Res. (2022) 27(1):51. 10.1186/s40001-022-00670-7 35379317 PMC8977183

[6] Liu RP Chen Y Wu HB Xiong FM He FY Li YY. Levels of fat-soluble vitamins A, D, E and their influencing factors in children with obesity. Chin J Contemp Pediatr. (2022) 24(5):572–8. 10.7499/j.issn.1008-8830.2111031 PMC9154372 35644199

[7] Yan BH Li Y Wu Q Gao NN Liu CY. Observation of vitamin D levels and analysis of influencing factors in 1,180 children aged 0–6 years in Jinan area. Shandong Med J. (2021) 61(9):27–30. 10.3969/j.issn.1002-266X.2021.09.007

[8] Huang D Qian X Wu T Zhu YX. Analysis of fat-soluble vitamin levels in children in the main urban area of Hangzhou. Chin J Matern Child Health. (2022) 37(19):3560–4. 10.19829/j.zgfybj.issn.1001-4411.2022.19.020

[9] Amimo JO Michael H Chepngeno J Raev SA Saif LJ. Immune impairment associated with vitamin A deficiency: insights from clinical studies and animal model research. Nutrients. (2022) 14(23):5038. 10.3390/nu1423503810.3390/nu14235038 36501067 PMC9738822

[10] Szymczak-Pajor I Miazek K Selmi A Balcerczyk A Śliwińska A. The action of vitamin D in adipose tissue: is there the link between vitamin D deficiency and adipose tissue-related metabolic disorders? Int J Mol Sci. (2022) 23(2):956. 10.3390/ijms23020956 35055140 PMC8779075

[11] Ungurianu A Zanfirescu A Nițulescu G Margină D. Vitamin E beyond its antioxidant label. Antioxidants. (2021) 10(5):634. 10.3390/antiox10050634 33919211 PMC8143145

[12] Varsamis NA Christou GA Kiortsis DN. A critical review of the effects of vitamin K on glucose and lipid homeostasis: its potential role in the prevention and management of type 2 diabetes. Hormones. (2021) 20(3):415–22. 10.1007/s42000-020-00268-w 33454929

[13] Kobylińska M Antosik K Decyk A Kurowska K. Malnutrition in obesity: is it possible? Obes Facts. (2022) 15(1):19–25. 10.1159/00051950310.1159/000519503 34749356 PMC8820192

[14] Wang X Xu M Li Y. Adipose tissue aging and metabolic disorder, and the impact of nutritional interventions. Nutrients. (2022) 14(15):3134. 10.3390/nu14153134 35956309 PMC9370499

[15] Nemati R McEntyre CJ Li BV Phillips I Sies CW. Fat-soluble vitamins: mechanisms and metabolism. A narrative review. N Z J Med Lab Sci. (2022) 76(1):3–8. https://irp.cdn-website.com/102112c1/files/uploaded/2022_-_Vol_76_No_1_%28March%29.pdf

[16] Weigand MC Schwandt EF Messersmith EM Levy AW Titgemeyer EC Blasi DA. 56 evaluating the seasonality effect of vitamin D status in commercial feedlot steers and heifers. J Anim Sci. (2025) 103:103211–212. 10.1093/jas/skaf102.231

